# Indian academy of pediatrics (neonatology chapter) recommendations for evidence-based neonatal skincare and protocols for hospitalized neonates

**DOI:** 10.3389/fped.2025.1433792

**Published:** 2025-06-12

**Authors:** Naveen Bajaj, R. Kishore Kumar, Arun Inamadar, Alok Bhandari, Rajesh Kumar, Kheya Ghosh Uttam, Jaikrishan Mittal, Pradeep Suryawanshi, Sanjay Wazir, Satyen Hemrajani, Priti Thakor

**Affiliations:** ^1^ Sapling Hospital, Ludhiana, India; ^2^ Cloudnine Group of Hospitals, Jayanagar, Bengaluru, India; ^3^ Department of Neonatology, Notre Dame University, Perth, WA, Australia; ^4^ Department of Dermatology, Venereology & Leprosy, Sri B. M. Patil Medical College, Hospital & Research Center, BLDE University, Karnataka, India; ^5^ Kukreja Hospital, New Delhi, India; ^6^ Rani Hospital and Rani Children Hospital, Jharkhand, India; ^7^ Pediatric Medicine Department, ICH, Kolkata, India; ^8^ Neoclinic, Jaipur, India; ^9^ Department of Neonatology, BVU Medical College, Pune, India; ^10^ Motherhood Hospitals, Gurgaon, India; ^11^ Mahatma Gandhi Medical College and Hospital, Jaipur, India; ^12^ Department of Medical Affairs, JNTL Consumer Health (India) Pvt. Ltd., Mumbai, Maharashtra, India

**Keywords:** intensive care units, neonatal, consensus, skin care, hospitals, emollients, parents

## Abstract

**Introduction:**

Infants in a neonatal intensive care unit (NICU) face a significant risk of skin barrier damage due to various factors related to their condition and the medical interventions they receive. The Neonatology Chapter of the Indian Academy of Pediatrics aimed to develop a comprehensive guidance document on evidence-based clinical practices for neonatal skin care in hospitalized infants, focusing on scientific principles and empirical evidence.

**Method:**

The modified Delphi method, involving a panel of 10 experts including neonatologists and pediatricians, was utilized to reach a consensus on clinical statements.

**Results:**

Out of 132 clinical statements formulated, 127 achieved consensus, while 5 did not. The experts advised implementing screening tools and risk assessment frameworks for skin injuries as part of the NICU's quality of care assessment. They emphasized the need for gentle cleansing of newborns to prevent skin or eye irritation and reduce the risk of skin infections. When determining the mode and method of skin cleansing in neonates, factors such as weight, gestational age (GA), and the severity of illness should be taken into account. Emollients may effectively prevent transepidermal water loss (TEWL) and be well-tolerated by NICU infants. Considering topical emollient therapy may significantly reduce mortality and hospital-acquired infections and improve weight gain. Multisensory stimulation in preterm infants has the potential to enhance feeding, psychomotor development, and visual function. Providing parents with a booklet that includes skincare regimens for cleansing the baby and protecting the skin barrier is essential for home care of their baby.

**Conclusion:**

This consensus aims to fill this void by offering clinical recommendations for the care of neonatal skin in hospitalized infants.

## Introduction

The skin, being the largest organ of the body, plays a crucial role in helping newborns adapt to life outside the womb (1). While structurally similar to adult skin, newborn skin differs in several key ways. It has a higher surface area to weight ratio (700 cm^2^/kg compared to 250 cm^2^/kg in adults), a weaker connection between the dermis and epidermis, thinner and less elastic properties, higher permeability in the stratum corneum, an underdeveloped epidermal barrier, reduced melanin production, and sweat glands that are dense but less active (2). Additionally, the skin of newborns and young infants tends to have a higher pH and lower free fatty acid content compared to adult skin (3). These distinctions render a newborn's skin more prone to injury and infection, necessitating specialized care. It is crucial to communicate certain principles of skin care to the mother or caregiver (2). Although prematurity is the primary reason for babies being admitted to a NICU, 42% of admissions involve term babies with certain complications (2).

Aside from the unique anatomy and physiology of immature skin, infants in NICUs face heightened risks of skin injuries due to the frequent need for procedures and essential invasive devices for their survival. Standard invasive procedures like venous or arterial punctures and the application or removal of adhesives can lead to skin rupture. Therefore, it is essential to implement evidence-based skin care practices and use appropriate products to enhance neonatal outcomes (3). These factors underscore the importance of providing meticulous care for neonatal skin and implementing appropriate measures for antisepsis. A recent study highlighted significant variation in bathing, asepsis, and skin care practices in Indian NICUs. The study found that 65% of NICUs did not use any scales or tools for skin assessment. Additionally, 90% of NICUs used sponging for hygiene, despite evidence favouring immersion/swaddle bathing. Only 14% of NICUs used wipes, while 50% expressed interest in using them, as studies suggest that wipes may aid in infection control and barrier repair. These discrepancies underscore the need for evidence-based guidelines for skincare and the use of antisepsis measures in Indian NICUs (4).

Studies indicate that the NICU environment can lead to abnormal sensory stimulation, with certain systems such as visual and auditory being overstimulated, while others like tactile and vestibular are under stimulated, which could disrupt brain development, particularly during the first year of life when critical periods for sensory system maturation occur. This sensory imbalance can significantly impact hospitalized neonates (5). Recognizing the existing need, the goal of organizing this expert group meeting was to establish evidence-based recommendations for practices and protocols in NICUs.

## Methods

### Process overview

A panel of 10 experts (neonatologists and pediatricians) participated in a Delphi process to identify consensus on about 10 domains. Importance of skincare in NICUs, neonatal skin assessment, skin hygiene in NICU babies, umbilical cord care, perineal care, TEWL and thermoregulation in NICUs: role of skin care in fluid and electrolyte balance, iatrogenic injuries, management of pressure injuries/wounds, neurodevelopmental care/developmental supportive care in NICUs and skin care regimen and parent education at discharge. Consensus was developed using the 3-step Delphi method, which took place between October 2022 and December 2022.

Initially, a thorough list of statements was formulated and distributed to an expert panel through an online platform for voting. Subsequently, a virtual meeting was conducted with the expert panel to discuss statements where consensus was not reached or which required further discussion. This allowed panel members to provide additional clarification on certain issues and present arguments to support their perspectives. Following this discussion, a second round of voting was conducted for the revised statements.

### Data extraction and statement development

Relevant information was extracted from a literature review and full-text publications focusing on best practices for assessing, caring for, treating, and providing skin care advice for hospitalized neonates, as well as best practices in neurodevelopmental care. This extracted information served as the basis for developing consensus statements. Members of the expert group reviewed these statements and convened as a group to discuss them (see Table 1). Panelists’ anonymity was preserved throughout the process, with all comments being incorporated anonymously into the statements and questionnaires distributed to panelists in each round.

**Table 1:** Delphi methodology and results.

Literature search
132 statements drafted
Round 1–132 statements circulated to panel members.
97 accepted without modification. 35 (13 did not reach consensus, and 22 needed further discussion) taken forward for discussion in round 2
Round 1 conclusion—Inclusion of 97 Statements in the Final Guideline Document
Round 2—Discussion of 35 Statements in the Advisory Board
5 statements omitted. 8 statements retained without modification. 22 statements modified for round 3 voting
Round 2 Conclusion—Inclusion of 8 Statements in the Final Guideline Document.
Round 3—Distribution of 22 Statements to Panel Members
All 22 statements—a consensus
End of Round 3: 22 statements incorporated in the final guideline document
Final Guideline document: 127 Consensus Statements (Combined from Round 1, Round 2, and Round 3)

#### Round 1

The initial draft document, comprising 132 statements, was distributed via email to all 10-panel members along with a detailed explanation of the study's objectives and specific instructions for participation. Each expert was requested to vote by indicating their level of agreement with each statement as “strongly agree,” “agree,” “neutral,” or “disagree.” Experts were also encouraged to provide comments and suggest additional items that may have been overlooked during the initial statement development. In round 1, the aim was to record the panel's agreement or disagreement with each statement and address any redundancies or issues related to the clarity or syntax of the statements. Response frequencies for each statement were calculated and entered anonymously into a database by a research assistant. Thirteen statements did not achieve 100% agreement, and 22 statements achieved agreement but required further discussion, which took place during a face-to-face meeting of the expert panel in round 2.

#### Round 2

During round 1, a total of 35 statements were identified, with 13 statements failing to achieve 100% agreement and 22 statements reaching agreement but requiring further discussion. All 10 members participated in a virtual face-to-face meeting to discuss these statements. Panel members were encouraged to discuss the remaining statements until a consensus was reached on whether to retain, modify, or eliminate them from the final guideline document. Consequently, five statements were removed, 22 were modified, and 8 were included based on the discussions. The revised 22 statements were then circulated to panel members for voting in round 3.

#### Round 3

In Round 2, 22 statements were revised and distributed to all panel members via email. Each expert was requested to vote by indicating their level of agreement with each statement as “strongly agree,” “agree,” “neutral,” or “disagree.” All 22 statements reached 100% agreement, and the final consensus arrived at 127 statements, which centered around primarily 10 domains viz. importance of skincare in NICUs, neonatal skin assessment, skin hygiene in NICU babies, umbilical cord care, perineal care, TEWL and thermoregulation in NICUs: role of skin care in fluid and electrolyte balance, iatrogenic injuries, management of pressure injuries/wounds, neurodevelopmental care/developmental supportive care in NICUs and skin care regimen and parent education at discharge (Table 2).

**Table 2:** List of final consensus statements to which 100 % agreement was obtained.

Section 1: Importance of skincare in NICUs	
1.	Neonates in a NICU are at high risk of skin barrier impairment due to intrinsic and extrinsic factors related to their condition and iatrogenic factors they may be exposed to during their admission to NICU (3).
2.	The incidence of skin injuries in term and preterm infants in NICU is vastly different in various settings and depends on the quality of assessment and care.
3.	NICU babies are vulnerable to thermal imbalance, fluid and electrolyte loss, skin injury, and sepsis arising from wound contamination and skin breakdown, all due to developmental immaturity of the skin (4).
4.	The stratum corneum is developing, resulting in a more significant TEWL and risk of microbial invasion (5).
5.	The body surface area to body mass ratio of newborns is more significant than that of children and adults, leading to increased water loss through the skin, large and fast absorption of drugs through the skin, and relatively apparent adverse drug reactions, which lead to increased mortality and morbidity (6).
6.	The risk of skin injuries and subsequent complications may be reduced if NICUs have established evidence-based skincare protocols and perform skin assessments as an integral part of management (3).
7.	The overall goal of skin care is to protect the skin barrier and prevent skin alterations, including skin injury and exposure to skin irritants and toxins, which may prevent morbidities such as dehydration and nosocomial infection (7).
8.	There is a broad variation in routine skin care practices in NICUs in India. Hence, evidence-based recommendations for standardized neonatal skin care practices in Indian NICUs are vital for improving neonatal outcomes (8).
9.	Skincare protocol integrated with core treatment of the condition in NICU is an essential aspect of Quality Improvement (QI) projects and should be implemented vigorously (9).
Section 2: Neonatal skin assessment	
10.	Assessment of neonatal skin surface from head to toe helps in the early identification and treatment of skin problems, identifying the risk associated with the skin integrity, and promptly detecting the need for more extended hospital stay (9).
11.	The use of screening tools and risk assessment frameworks for skin injuries should be a part of the assessment of the quality of care in NICU (9).
12.	Teams working in the NICU should be equipped to detect skin injury in neonates and differentiate between transient benign skin lesions vs. more severe cutaneous findings (10).
13.	Skin assessment should be done using a valid and reliable assessment tool that can determine the current skin condition and risk of injury/breach (11).
14.	Skin assessment should be performed routinely and, if possible, in every shift (11).
15.	Neonatal Skin Condition Score (NSCS) is a skin assessment tool that can be easily used by nursing staff but does not substitute predictive risk assessment tools in neonates at high risk of developing skin injuries (9, 12). *Note: NSCS is a valid and reliable assessment tool that can be used to evaluate skin conditions from very-low-birth weight to full-term neonates by single and multiple raters to assess neonatal skin condition, even across weight groups and racial groups. This 9-point scale measures dryness (1–3), erythema (1–3), and breakdown (1–3). Best possible score: 3; worst possible score: 9. If a neonate scores a single score of 3 in one area or a combined score of *≥*6, the relevant medical team must be notified, and an action plan is to be documented in the patient’s progress notes. A dermatology referral may also be appropriate.*
16.	The application of reliable pressure ulcer risk assessment tools provides a valuable means for the identification of risk levels and types of pressure injury in newborns so that appropriate and timely prevention strategies can be implemented (9, 13). *Note: Pressure Ulcer Risk Assessment scales studied in neonates are– Braden Q, Braden QD, Glamorgan Scale, Starkid and Neonatal Skin Risk Assessment Scale (NSRAS), Skin Risk Assessment and Management Tool (SRAMT)*
17.	The Braden Q scale is a validated pressure ulcer risk assessment tool and has moderate predictive validity with medium sensitivity and low specificity for pressure ulcers in NICUs (9, 14). *Note: Braden Q Scale assesses risk for pressure sores and skin breakdown in seven categories (sensory perception, moisture, activity, mobility, nutrition, friction and shear, and tissue perfusion–oxygenation) with scores from 1 to 4 for each with scale anchors varying with each category. Highest risk: 7; Lowest risk: 28. Score <16 implies a risk for pressure ulcers. There are several weaknesses in the Braden Q Scale, including an inability to identify medical device-related pressure injuries. Additionally, the scale was initially applied to children aged 3 months to 8 years and was inapplicable to patients with congenital heart disease.*
18.	The Braden QD Scale is an improvement over the Braden Q (15). *Note: It can predict pressure injury risk due to immobility and the use of medical devices. This scale was examined based on participants ranging from preterm age up to 21 years old and those with various medical conditions. *
19.	The Modified Glamorgan Pediatric Pressure Injury Risk Assessment Scale (2012) is more effective as it predicts the level of risk of pressure injury and provides suggested interventions as per the level of risk (9, 16). *Note: This tool was developed and validated specifically for use in children from birth to 18 years. It considers mobility and devices. At risk: 10+; high risk: 15+; very high risk: 20+. Pressure injury risk is re-assessed daily in inpatient areas and every time there are significant changes in the child's condition.*
20.	SRAMT has a higher predictive value than Braden Q in predicting neonates at risk of injury and is hence preferred in NICUs. *Note: The tool is composed of 3 sections; risk assessment, care protocol and management guidelines* (3, 9)*.*
21.	Predicting neonates at risk of skin injury will only reduce injury rates if supported by skin care policy, guidelines, and vigilance in daily care (3).
22.	NSCS is a good tool for routine assessment. In cases where the NSCS score indicates higher risk, SRAMT can be used (9, 12). *The statement is based on the expert panel evaluation of the tools and recommendation for use in clinical practice
Section 3: Skin hygiene in NICU babies	
23.	Skin hygiene, including utmost care of their skin and appropriate antisepsis measures, is vital for reducing infection in neonates and hence helps reduce the incidence of neonatal sepsis (8).
24.	Stringent hand hygiene practices in the NICU shall reduce the risk and incidence of neonatal infections and ultimately contribute to the desired reduction in infection-related neonatal deaths (17).
25.	Hand washing is recommended to be performed before touching hospital equipment and instruments, before handling neonates, and in between cleaning and caring for neonates (17, 18).
26.	Alcohol-only and alcohol-plus chlorhexidine hand sanitizers could be used for hand sanitization as both are effective against some commonly found hospital pathogens (19).
27.	Cleansing of newborns needs to be carried out with particular care to avoid skin or eye irritation and predisposition to skin infections (7).
28.	As neonates could be considered contaminated with blood-borne pathogens until they are cleansed after birth, removal of blood and secretions may help to minimize the risk of infections (9).
29.	Vernix Preservation and natural absorption should be encouraged (9).
30.	For neonates, provide the first bath once the neonate has achieved cardiorespiratory and thermoregulatory stability (20).
31.	Skin-wiping with chlorhexidine wipes (up to 2%) once at the time of admission to NICU may be done to reduce colonization (in high-risk settings, e.g., extramural babies) in those who are at high risk of sepsis or infection (20).
32.	Consider weight, GA, and severity of illness while deciding on the mode and method of skin cleansing in neonates (20).
33.	Neonates with GA less than 28 weeks should not be bathed, and in case of soiling, sterile pre-warmed water could be used to cleanse with gentle patting of the skin to dry (21). Neonates with GA 28-32 weeks may be sponged with sterile lukewarm water. Stable babies >32 weeks may be bathed using the swaddled immersion technique.
34.	Alternatively, cleansing with appropriately formulated wipes and mild cleansing agents that remove bacteria and moisturize the skin may be considered (22). (a) Appropriately formulated baby wipes contain extremely mild surfactant (detergent or cleanser) to lower surface tension for better cleaning, a preservation system to ensure no microbial growth, a pH adjusting (buffering) system to maintain pH like neonatal skin, and skin-benefiting ingredients including emollients (23).
35.	For bathing during the first week of life, consider the use of sterile warm water only for neonates <32 weeks of gestation. Add a few drops of pH-neutral or slightly acidic cleansers for neonates >32 weeks of gestation (20).
36.	Babies in NICU, especially those with low to very low birth weight, are vulnerable to nosocomial infections, such as those caused by *Staphylococcus aureus* (9)*.*
37.	Consider using topical antiseptic agents in neonates before any intervention for the prevention of healthcare-associated infections (9).
38.	(a) Parents are advised to bathe themselves daily prior to kangaroo care (24). (b) In cases where daily bathing may not be possible, appropriately formulated wipes may help (24).
39.	Povidone Iodine should be avoided for the risk of thyroid suppression (25).
40.	Infection from medical devices during any invasive procedure is one of the most significant risks for neonates in NICU (9).
41.	The skin surface of the neonates should be disinfected before any surgical procedure is done using medical devices (9).
42.	Using antiseptic solutions as disinfectants for skin surfaces before any invasive procedures (central venous catheter insertion, catheter site maintenance, umbilical line insertion, and peripheral venous line insertion) reduces the risk of infections (26).
43.	Ensure the efficacy and short- and long-term safety of disinfectants before using term and preterm neonates, and remove all disinfectants altogether from the skin with sterile water or saline after the procedure (9).
Section 4: Umbilical cord care	
44.	Dry cord care continues to be the most appropriate approach in settings with a low incidence of omphalitis (9). *Note: Dry cord care consists of cleansing the stump with tepid water and a mild cleanser and keeping the cord dry. Additional measures have been proposed for this approach, such as covering the stump with clean gauze, exposing it to air by keeping it outside the diaper or avoiding its immersion in water.*
45.	Routine use of chlorhexidine is indicated in regions or settings with a high incidence of omphalitis and associated neonatal death (9).
Section 5: Perineal care	
46.	Perineal care involves cleaning the genital and rectal areas of the neonate and should be done routinely to avoid infection and sepsis (27).
47.	Diaper dermatitis (DD) causes discomfort and emotional distress and creates possible sources of infection among NICU infants (27).
48.	Superabsorbent diapers are recommended as they trap urine and stool to prevent over-hydration while sequestering stool away from the skin (9).
49.	Baby wipes (preferably disposable) could be used to clean neonatal skin by removing fecal material and are designed to positively impact skin physiology, skin barrier function, and skin pH (28).
50.	Appropriate emollients may be used prophylactically to protect the skin from external irritants (29).
51.	Barrier creams (Petrolatum/Zinc) should be used on all neonates at every diaper change at the first sign of erythema or skin breakdown (20).
52.	Correct diagnosis of DD and classification into contact dermatitis or candida albicans-associated dermatitis is critical for management (30).
53.	Consider using zinc oxide cream or petroleum jelly over barrier cream in neonates with contact irritant dermatitis (9).
54.	For candida albicans-associated dermatitis, antifungal ointments should be considered as a barrier to the prevention of skin breakdown (9).
Section 6: TEWL and thermoregulation in NICUs: role of skin care in fluid and electrolyte balance	
55.	Evaporation loss through the skin (TEWL) usually contributes to the majority of Insensible Water Losses (IWL) in NICU babies (31).
56.	The emphasis in fluid and electrolyte therapy should be on the prevention of excessive IWL using single/combination techniques to minimize evaporative heat loss rather than replacement of increased IWL (9).
57.	Thin, transparent plastic barriers (e.g., cling wrap) should be used to increase the local humidity, decrease heat losses, limit air movement, and avoid excessive IWL (32).
58.	Usage of emollients could be practical (in preventing TEWL) and tolerable in NICU babies (33).
59.	Topical emollient therapy may be considered as it may reduce mortality and hospital-acquired infections significantly and improve weight. (Especially in low-resource settings) (34).
60.	Emollients like sunflower seed oil, coconut oil, or mineral oil can be considered for use in NICUs (20).
61.	A thin layer of emollient should be applied to the skin after cleansing (20).
Section 7: Iatrogenic injuries	
62.	Use of medical devices (respiratory, invasive ventilation, monitoring), medical condition (GA, low birth weight, immobility, comorbidities, skin immaturity), length of hospital stay, and care practices are some of the risk factors for iatrogenic pressure injury formation in neonates (35).
63.	Neonates born before 32 weeks of gestation are at the most significant risk of iatrogenic skin injury (36).
64.	Neonates with impaired gas exchange, tissue perfusion from cardiorespiratory disease, anemia, hypotension, and ventilation support are also at risk of skin injury (37).
65.	Nutritional control is necessary because low birth weight, weight loss after 4–5 days of birth, malnutrition, and dehydration may contribute to the development of Pressure ulcers in neonates (38).
66.	The skin under medical devices (nasal continuous positive airway pressure (CPAP) prongs and masks, vascular catheter hubs, central line interfaces, arm boards, tracheostomy tubes, and plaster cast edges) should be checked regularly every 12 h or more frequently, as required (9).
67.	The supporting surface is considered one of the medical device-related risk factors for pressure injury (39).
68.	The supporting surface should be assigned based on age, risk, body surface area, presence or absence of pressure injury, pressure injury severity, and baseline pathology of neonates (40). The occipital area of the neonate must be highly protected compared to the other areas of the body, as this zone is the one that maintains more pressure in neonates (40).
69.	Adult mattresses and linen mattresses should not be used for neonates as they are not suitable for their unique morphology (40).
70.	Consider using water mattresses, air-filled mattresses, gel mattresses, air alternating pressure relief mattresses, pressure relieving mattresses (mattress for cooling), or pressure diffusing mattresses (Giraffe warmer, warmer gel) for cooling or reducing the pressure sores or skin injuries in neonates (9, 11).
71.	In the NICU, the position of the neonates and installed devices must be changed frequently by nursing staff, and the positioning changes must be documented (41).
72.	Various positioning aids could be helpful to prevent pressure injury when applied to the pressure points (especially scalp, head, and occiput), including gel wedges, positioning bolsters, gel protectors, fat pads, ear pads, and com feel (11).
73.	Healthcare professionals must be trained in the use of repositioning equipment (42).
74.	The use of probes in various monitoring equipment or medical devices (including endotracheal tube, CPAP, ventilator, etc.) could be a risk factor for pressure injury in neonates. Hence, continuous rotation of probes with documentation is vital to prevent pressure injuries (9, 42).
75.	Resite probes every 24 h, and do not place them underneath the infant (43).
76.	All the rotation details for the probes in the neonate body should be documented (44).
Section 7 a: Adhesives	
77.	Neonates are always at high risk for medical adhesive–related skin injuries (MARSI) as neonatal skin is thinner than the skin of an adult, with an underdeveloped stratum corneum (42). *Note: Mechanical problems, including skin stripping, skin tears, and tension blisters; dermatitis reactions such as irritant contact dermatitis and allergic dermatitis; and other complications such as skin maceration and folliculitis are some of the MARSIs*
78.	Medical adhesives should be used with extreme caution. Adhesives that secure medical devices with less trauma should be selected (9).
79.	Medical adhesives should be chosen on the basis of their intended purpose, anatomic location, and ambient conditions of the skin site (9).
80.	Leukoplast tapes are to be avoided, especially in neonates <27 weeks GA (57).
81.	Less damaging types of adhesives are hydrogel, silicone-based, or hydrocolloid (9). *Note: Hydrocolloids cause similar skin trauma as acrylates and increase TEWL and erythema, but they absorb moisture, mold well to skin surfaces, and serve as a base layer for other adhesives* *Hydrogels have a highly breathable adhesive property with low trauma and provide a cooling or analgesic effect to wounds; however, they absorb exudate and may dislodge* *Silicone-based adhesive products have suitable adherence properties with less trauma, but they do not adhere well to plastic devices.*
82.	Consider using silicone-based skin protective films between perilesional skin and adhesive dressing when appropriate (39).
83.	When fixing medical devices, use a fixation method that avoids displacement and does not add additional pressure to the skin (44).
84.	Silicon-based adhesive tapes could be used on neonates’ skin to secure medical devices in place. Standard acrylate adhesive tapes may be used alternatively (57).
85.	Medical adhesives should be removed slowly and carefully in a horizontal direction using warm water with soft paraffin, moistened gauze, or saline pledgets at least 24 h after application (57).
86.	To remove dressings, if available, use silicone-based adhesive remover; otherwise, use distilled water (57).
87.	Apply mineral oil or petrolatum to loosen the tape unless retaping is necessary at the site (9).
Section 7b: Intravenous extravasation	
88.	It is essential to identify different factors that increase the neonatal risk for intravenous extravasation as it is associated with significant morbidity (9).
89.	A site assessment should be conducted every hour when fluids or medications are running through the line. If nothing is being infused, the site should be assessed before accessing the line and at least every 8 h (43).
90.	If a grade 3/4 injury in a community setting is reported, the medical team needs to be notified immediately, and treatment, if required, should be commenced within 1–2 h (44).
91.	The risk of extravasation could be minimized with (1) use of plastic vascular access devices, (2) selecting veins in the most distal part of the extremity, (3) avoiding placing vascular access devices in areas challenging to immobilize (if possible), (4) securing vascular access devices with transparent adhesive dressings, (5) avoiding positioning the tip of the cannula over a joint or lateral malleolus (6) removing any cannulas that are not being used (9).
Section 8: Management of pressure injuries/wounds	
92.	The potential causes of skin injury should be carefully evaluated (adhesive removal, warming devices, irritant solutions, infection, and thrombolytic events) to plan a specific management strategy as per the stage or status of the wound (9).
93.	The choice of treatment must depend on the GA at birth, pressure ulcer category, location, risk of infection, skin type, and pathology (39).
94.	Understanding the principles of moist wound healing, gentle cleansing, and prevention of mechanical trauma, as well as choosing and correctly applying the appropriate dressing, leads to better outcomes for neonatal skin injuries (9).
95.	If prescribed, use antibacterial ointment or antifungal ointment that is targeted to the skin injury. However, systemic treatments are only considered for the high-risk groups, including very low birth weight (VLBW) and extremely low birth weight (ELBW), with clinical instabilities (9).
96.	Normal isotonic saline, either diluted with sterile water (1:1) or undiluted, could be used to clean the affected area. Avoid the use of antiseptic skin cleansers, such as hydrogen peroxide, povidone–iodine, iodophor, Dakin’s solution, acetic acid, and any alcohol-containing antiseptic, even diluted (9).
97.	Use appropriate, occlusive, and non-adherent dressings as per the stage of wound healing. Avoid dressings that result in skin stripping and peri-wound injury (9).
98.	Silicone-based adhesives, hydrocolloids, polyurethane films, hydrogels, or silver dressings could be used for wounds or large denuded areas (9).
Section 9: Neurodevelopmental care/developmental supportive care in NICUs	
99.	The increasing survival rates of preterm infants admitted to the NICU have emphasized the importance of considering their neurodevelopmental outcomes, as there is a risk of significant neurodevelopmental delays (45).
100.	Environmental stimulation (i.e., light, noise), social interactions with parents, and caregiving experience are among influential environmental NICU factors that influence neurodevelopmental outcomes (46).
101.	Sensory stimulation is an intervention that, through peripheral stimuli, can facilitate brain organization due to neuronal plasticity and cortical reorganization (47).
102.	Family-integrated, neuroprotective, developmentally supportive care to be incorporated (48). *Note: The NIDCAP (Newborn Individualized Developmental Care and Assessment Program) model focuses on a detailed reading of each infant’s behavioral cues. These cues dictate the environmental and care adaptations that are required to support and enhance each infant’s strengths and self-regulation capacities.* *The Neonatal Integrative Developmental Care Model (IDC) identifies seven distinct core measures that provide clinical guidance for NICU staff in delivering neuroprotective family-centered developmental care to preterm infants and their families in the NICU.*
103.	Multisensorial stimulation can form an integral part of neurodevelopmental or Developmental supportive care (47).
104.	Multisensory stimulation in preterm infants may improve feeding, psychomotor development, and visual function (47). *Note: Multisensory stimulation is described as an intervention that uses stimuli in more than one sensory category, in any combination: kinesthetic, tactile, visual, gustatory, olfactory, auditory, and vestibular.* *The most used stimuli in studies are auditory, tactile, visual, and vestibular stimuli.*
105.	Scientific evidence supports the NIDCAP model, which encompasses skin-to-skin holding and care (46).
106.	Activities of daily living can optimize opportunities for sensory stimulation: cleansing and tactile stimulation (49).
107.	Kangaroo Mother Care (KMC) is a simple method of care that includes early and prolonged skin-to-skin contact with the mother (or a substitute caregiver) and exclusive and frequent breastfeeding (50).
108.	The defining feature of KMC is direct contact between parental skin and infant skin by holding a diaper-clad infant on a parent's bare chest in an upright, prone position (51).
109.	Facilitate early, frequent, and prolonged KMC by encouraging zero separation between parents and infants (52).
110.	While there are no standard guidelines for the duration, the most common treatment duration studied for KMC is 10–15 minutes twice a day for 15 days (47).
111.	Provide a comfortable and safe reclining chair or adult bed for early, frequent, and prolonged KMC (53).
112.	NICUs should be designed to encourage family reunification and presence, facilitate psychosocial support, address/minimize sensory impact, offer social connection, and enable positive parental experiences (54).
113.	Focus on facilitating sleep and minimizing stress in NICU babies (54). *Note: At approximately 28 weeks gestation, individual sleep patterns begin to emerge characterized by rapid eye movement (REM) and non-rapid eye movement (NREM) sleep periods. REM and NREM sleep cycling are essential for early neurosensory development, learning and memory, and preservation of brain plasticity.* *Minimizing stress in preterm infants has many neurologic benefits, such as reducing the likelihood of programming abnormal stress responsiveness, which will help preserve existing neuroplastic capacity.*
114.	Positioning (including nesting) should be considered to promote quality sleep and decrease arousal from sleep. Preterm infants are more likely to remain sleeping when they are in a prone position (54).
115.	To consistently manage stress and pain in neonates, accurate monitoring of discomfort, as the “fifth” vital sign, needs to be assessed utilizing a standardized pain assessment tool (54).
116.	For standard painful procedures, such as heelsticks, venipunctures, and orogastric tube (OG) insertions, non-pharmacological interventions such as multisensory stimulation should be the first choice in non-compromised infants (54).
Section 10: Skincare regimen and parent education at discharge	
117.	Since the immune system of babies who have spent time in NICUs is still maturing, they are more prone to infections, and a strong skin barrier can protect them (7).
119.	Skincare regimens to cleanse the baby and protect the skin barrier must be a part of the booklet provided to parents for caring for their baby at home (52).
120.	Post-discharge for the first week, cleansing may be done using appropriately formulated wipes. A thin layer of emollient may also be applied (20). *The statement is based on an expert panel evaluation of the literature and recommendation for use in clinical practice
121.	Swaddle immersion bathing is effective in terms of control of body temperature and motor activities to decrease behavioral stress. However, make sure to disinfect bathing equipment appropriately (9).
122.	Sponge bathing may lead to heat loss and behavioral stress apart from barrier compromise due to the friction (9).
123.	An appropriate skincare regimen may facilitate average commensal growth on the skin and help improve immune system maturation (9).
124.	Mothers should be trained to assess the skin for any sign of barrier damage and immediately seek medical attention (9).
125.	Do’s and Don’ts of skincare should be part of the discharge booklet (55).
126.	Once the baby is adapted to transition to home, a skincare regimen that incorporates multisensorial stimulation should be followed: massage, bath, and emollient use *The statement is based on an expert panel evaluation of the literature and recommendations for use in clinical practice (56).
127.	Parents should be encouraged to bathe their babies as bath time is an excellent opportunity for parent-infant interaction. Parents should talk to their infant and engage their infant in play activities (9).

## Discussion

The fragile nature of newborn skin makes it prone to injury and infection, particularly in NICU neonates, necessitating specialized care to mitigate harm and promote optimal skin development (6). However, existing guidelines often overlook the unique challenges of NICU skin care, highlighting the need for evidence-based recommendations. This expert consensus on neonatal skin management in the NICU aims to align with evidence-based practices and enhance routine skin care. It also focuses on preventing and treating iatrogenic skin injuries in neonates, ultimately leading to improved patient outcomes.

Tools like the NSCS and SRAMT aid in skin assessment, with the latter being preferred for high-risk neonates (7). The experts have underscored the importance of preserving neonatal skin integrity and preventing skin injuries in the NICU. Strategies include using topical emollients to strengthen the skin barrier, thereby reducing the risk of invasive infections (8). Emollients such as sunflower seed oil, coconut oil, or mineral oil can help prevent TEWL and lower the risk of mortality and infections in NICU settings (9, 10).

Due to their thinner and less developed skin, neonates are highly vulnerable to medical adhesive-related skin injuries (MARSI), which can include mechanical issues and dermatitis reactions (11). Thus, cautious selection of less damaging adhesives, such as hydrogel, silicone-based, or hydrocolloid types, is crucial while avoiding leukoplast tapes and using fixation methods that minimize skin pressure and displacement of devices. Research suggests that newer formulations of silicone-based adhesives could be less disruptive to the skin barrier than acrylate adhesive tapes, and may also adhere to plastic, and could be used for tubing securement devices (7, 12).

Research suggests a possible connection between neonatal exposure to iodine-containing disinfectants and thyroid dysfunction in infants born before 32 weeks. This highlights the critical need for careful practices when using chlorhexidine in the NICU environment, also before any invasive interventions (13). Additionally, preventing pressure ulcers in neonates necessitates a comprehensive approach to skincare, incorporating hygiene, moisture control, and pressure management, with particular attention to nutritional support (14).

Emphasizing skin hygiene and meticulous care, including hand hygiene, can reduce infections and sepsis (15). On the other hand, maintaining proper skin care practices, including perineal care and the use of superabsorbent diapers and barrier creams, is crucial for promoting skin health (7). Maintaining the proper pH balance for the skin is crucial for both immediate skin health and long-term well-being. Newborn infection protection relies on staff and parent hygiene, as well as routine disinfection of catheter insertion sites and aseptic handling of central venous catheters.

Additionally, strategies to minimize IWL and prevent iatrogenic pressure injuries are crucial in the NICU setting. Careful assessment and management of neonatal skin injuries by health care physicians at each of the NICU visits, along with environmental factors like sensory stimulation and family-integrated care, are essential for optimal neurodevelopmental outcomes (16). Integrating these practices into routine care and providing education to parents can enhance the well-being of NICU infants and improve overall outcomes (58).

Babies from the NICU need strong skin barrier protection and skincare education for parents, including proper cleansing and emollient use, outlined in discharge materials. Cleansing approaches should focus on swaddle immersion bathing and avoid sponge bathing to reduce heat loss and stress, promote healthy skin flora, and involve parents in skincare routines (7).

There is a further need to develop an appropriate customized recommendations for stratified gestational age in neonates, while also considering complexities of the profiles under consideration. These would include benefits over both the short-term, and long-term (hydration, TEWL, skin pH, maternal satisfaction, skin colonization, erythema, etc.).

Following these expert consensuses and their clinical recommendations for neonatal skin care in hospitalized neonates may ultimately lead to improved patient outcomes. Adherence to these guidelines ensures comprehensive and effective management of neonatal skin conditions, promoting the well-being of newborns in hospital settings. Further research on the management of sensitive skin in the pediatric population is warranted.
