# Correction: Indian academy of pediatrics (neonatology chapter) recommendations for evidence-based neonatal skincare and protocols for hospitalized neonates

**DOI:** 10.3389/fped.2025.1667273

**Published:** 2025-07-29

**Authors:** Frontiers Production Office

**Affiliations:** Frontiers Media SA, Lausanne, Switzerland

**Keywords:** intensive care units, neonatal, consensus, skin care, hospitals, emollients, parents

The wrong email for the corresponding author was listed. The correct email for the corresponding author is docprit@gmail.com.

The original version of this article has been updated.

